# Long-term clinical results of early-stage lung cancer patients treated with risk-adapted stereotactic body radiotherapy using LINAC or CyberKnife: A single-institution analysis of more than 400 cases

**DOI:** 10.1007/s00066-025-02455-3

**Published:** 2025-08-25

**Authors:** Zsolt Levente Jánváry, András Bajcsay, Gábor Stelczer, Gábor Kontra, Tamás Pócza, Mercédesz Gerdán, József Lövey, Zsuzsa S. Kocsis, Katalin Ladányi, Éva Pap, Tibor Major, Csaba Polgár

**Affiliations:** 1 Center of Radiotherapy, National Institute of Oncology, Budapest, Hungary; 2 Department of Nuclear Techniques, Institute of Nuclear Techniques, Budapest University of Technology and Economics, Budapest, Hungary; 3 Department of Oncology, Oslo University Hospital, Norway, Oslo, Norway; 4 Department of Radiobiology and Diagnostic Onco-Cytogenetics, Center of Radiotherapy, National Institute of Oncology, Budapest, Hungary; 5 Department of Oncology, Semmelweis University, Budapest, Hungary; 6 National Tumor Biology Laboratory, National Institute of Oncology, Budapest, Hungary

**Keywords:** Stereotactic body radiotherapy, Lung cancer, Stereotactic ablative radiotherapy, Risk-adapted radiotherapy, Image guided radiotherapy

## Abstract

**Purpose:**

The aim of the study was to evaluate the clinical efficacy and side effects of stereotactic body radiotherapy (SBRT) using a gantry-based linear accelerator (LINAC) or robotic technique in a large cohort of consecutively treated medically inoperable early-stage lung cancer patients.

**Methods:**

Between March 2015 and February 2023, 401 early-stage (T1–2 N0 M0) primary lung cancer patients were treated using either LINACs (Varian VitalBeam® and TrueBeam®; Varian, Palo Alto, CA, USA) or CyberKnife® (Accuray, Madison, WI, USA). Median age was 70 years (range 44–90). Diagnosis was based on biopsy for 37.4% of patients, while pathological confirmation was unavailable due to high risk for 62.6%. ^18^F‑fluorodeoxyglucose positron-emission tomography (18-FDG-PET) was part of the pretreatment diagnostic workup in 96% (*n* = 386) of the total cohort. Tumor stage distribution was T1a in 32 (8%), T1b in 179 (44.6%), T1c in 112 (27.9%), T2a in 67 (16.7%), and T2b in 11 (2.7%) patients. Applied dose schemes were identical for both LINAC and CyberKnife treatments, using risk-adapted doses of 45–60 Gy in 3 to 8 fractions, (biologically effective dose ranging from 86 to 151.2 Gy BED_10_).

**Results:**

At a median follow-up of 32 months (range 2–104), the crude survival rate was 58%. Median overall survival (OS) was 63 months (95% CI: 51.1–74.8) the 2‑, 3‑, and 4‑year OS rates were 79, 68, and 56%, respectively. Actuarial local control (LC) rates were 94% at 2 years, 90% at 3 years, and 87% at 4 years. Median LC was not reached. Median local progression-free survival (LPFS) and progression-free survival (PFS) rates were 49.5 months (95% CI: 42.8–56.3) and 37 months (95% CI: 31.2.–42.8), respectively. Actuarial 2‑, 3‑, and 4‑year LPFS and PFS rates were 75, 60, and 51% and 66, 51, and 42%, respectively. On multivariate analysis, BED_10_ ≥ 132 Gy predicted improved LPFS, while earlier tumor stage and better ECOG performance status were associated with improved OS. No grade 3 or higher acute side effects were observed. Grade 3 late side effects occurred in 4 patients (1%), including grade 3 late pulmonary fibrosis in 3 cases and potentially treatment-related grade 3 pneumothorax in 1 patient. Rib fracture was observed in 14 cases (3%).

**Conclusion:**

Clinical results after SBRT at a national comprehensive cancer center demonstrate high LC and LPFS rates and favorable PFS and OS, comparable to published studies. Application of a BED_10_ of 132 Gy or higher shows a potential benefit in terms of LPFS and may thus be recommended in the absence of conflict with organ at risk constraints. SBRT with either LINAC or CK is proven to be a well-tolerated but still highly effective treatment for the elderly, medically inoperable early-stage lung cancer population.

**Supplementary Information:**

The online version of this article (10.1007/s00066-025-02455-3) contains supplementary material, which is available to authorized users.

## Introduction

Lung cancer is one of the most frequent malignancies worldwide, with more than 2 million new cases per year [1]. Hungary is reported to have one of the highest incidences amongst European countries, with more than 9500 newly diagnosed lung cancer patients on a yearly basis [2]. Early diagnosis (stages T1–2 N0 M0) is established in only 16–20% of these patients [3, 4]; however, screening programs can help to further increase this proportion.

The gold standard treatment of early-stage non-small cell lung cancer (NSCLC) is surgical removal, but a significant proportion (up to 25%) of patients are ineligible for surgery because of poor lung function, severe comorbidities, or older age [5]. In the case of medical inoperability or, more rarely, refusal of surgery, stereotactic body radiation therapy (SBRT) offers a curative treatment option by having the potential to deliver higher biologically effective doses than conventional radiotherapy techniques. Solid evidence on a direct comparison between surgery and SBRT is still missing, since earlier studies (STARS, Dutch ROSEL) were prematurely terminated for insufficient accrual. However long-term revision of the STARS trial in a propensity-matched comparison has shown lung SBRT to be non-inferior to video-assisted surgery in operable stage IA NSCLC [6].

The long-term results of ongoing randomized trials are eagerly awaited on this subject (VALOR, NCT02984761; STABLE-MATES, NCT01622621). On the other hand, the role of SBRT in inoperable patients is strongly established in the literature, supported by international guidelines such as those from ASTRO (American Society for Radiation Oncology), ESTRO (European Society for Radiotherapy and Oncology), and EORTC (European Organisation For Research And Treatment Of Cancer) [7–9], and this approach is applied in many centers worldwide.

Local control (LC) rates of lung SBRT treatment are outstanding, ranging between 77 and 96% at 3 years [10–13]. Overall survival (OS) rates are reported to be between 55 and 87% at 3 years [10, 12–16], while the rate of severe late toxicities is low (0–4%) [10–15]. For a substantial proportion of inoperable patients, comorbidities contraindicate any invasive histological confirmation. For those with clinical diagnosis only (based on CT morphology and progression or high SUV uptake on 18-FDG-PET/CT), SBRT has been also validated as a curative approach [13].

In this retrospective, single-center study, we aim to analyze the long-term clinical results, prognostic factors, and safety of a large cohort of early-stage primary lung cancer patients treated with stereotactic body radiotherapy in a tertiary referral oncology center.

## Materials and methods

Pulmonary SBRT was implemented in the National Institute of Oncology (NIO) in Budapest in 2015 using a gantry-based linear accelerator (LINAC) technique, and the CyberKnife (CK; Accuray, Madison, WI, USA) robotic respiratory-tracking method was introduced in 2018. From that time on, both procedures were used in parallel. Receiving radiotherapy patients from several hospitals, even from the countryside, the NIO performs the highest number of SBRT treatments in Hungary. After ethics board approval from the Hungarian Medical Research Council, we retrospectively assessed the data of patients treated with lung SBRT using a LINAC or the CK between March 2015 and February 2023. Patients with previous pulmonary radiotherapy, presenting with local recurrence after lung surgery, harboring lesions greater than 5 cm, or suffering from lung metastasis from other primaries were not included in the current study. At the time of analysis, the interval from the RT start date should have been longer than 12 months. Altogether, data of the first 401 consecutive patients with stage T1–2 N0 M0 primary lung tumors treated with curative-intent SBRT were analyzed.

Before treatment, all cases were discussed in thoracic multidisciplinary tumor boards, and the decision for SBRT was overwhelmingly made due to medical inoperability. Histological confirmation was recommended for patients to be treated with SBRT, but in cases of  high risk for invasive biopsy, strong clinical diagnosis based on 18-FDG PET/CT positivity was accepted as well.

Treatment preparation and motion-management protocols were adapted in terms of the specifications of the two available RT techniques, i.e., LINAC or CyberKnife® (CK).

### SBRT procedure for LINAC

To manage respiratory motion, patients receiving gantry-based treatment underwent four-dimensional (4D) CT simulation (Somatom Definition AS Open and Somatom Go Pro; Siemens, Munich, Germany) with 2 mm slice thickness using wingboard immobilization and tattoo skin markings. Abdominal compression was applied using a pneumatic compression belt (Civco, Coralville, IA, USA) in cases with lower-lobe lesions in order to decrease tumor motion. Target volume definition was based on the internal target volume (ITV) method, starting with the union of gross tumor volumes (GTVs) contoured for the macroscopically visible tumor on lung windowing in 7–10 respiratory phases, encompassing the full range of motion. The accumulated GTV (GTV Acc) was expanded by a 3-mm margin to achieve the clinical target volume (CTV), which was manually corrected for bones or mediastinal organs, then a 2-mm margin was added for the planning target volume (PTV). The Eclipse (Varian, Palo Alto, CA) treatment planning system (version 13.6 or higher) was used for dose calculation (Acuros® XB algorithm). LINAC treatments were delivered using the volumetric modulated arc therapy (VMAT) technique, with 6 MV energy in flattening filter-free (FFF) mode on either a Varian VitalBeam® equipped with a four-degrees-of-freedom (4DoF) couch or using TrueBeam® machines equipped with a 6DoF couch (Varian® Medical Systems, Palo Alto Ca, USA). All treatments with abdominal compression were carried out on the TrueBeam machines. Image-guided radiotherapy (IGRT) was performed with daily cone-beam CT (CBCT) to correct any setup errors prior to treatment.

### SBRT procedure for CyberKnife

For patients to be treated with CyberKnife, a session of presimulation was performed to verify whether direct kilovoltage tumor motion tracking (Xsight Lung Tracking System; Accuray) could be performed, in order to omit invasive gold marker placement. The Xsight Lung respiratory tracking option can be applied if the tumor is detectable on both (2-view), or at least one (1-view) of the orthogonal X‑ray IGRT images acquired by the CyberKnife system. Direct tumor tracking can be performed for such visible tumors by registration of the tumor in digitally reconstructed radiographs (DRRs) generated from the planning CT image to the corresponding region in the treatment X‑ray images. The different tracking options of the CK system, including Xsight Lung, have been described in detail in other studies [15, 17–19].

The CT simulation was performed according to the specific recommendations for the CK systems, with acquisition of exhale and inhale breath-hold CT image sets with 1.25 mm slice thickness in supine position with arms alongside the body. Target volume definition was as follows: for 2‑view cases, the GTV was contoured only on exhale phases and extended by 3 mm to achieve the CTV, which was manually corrected for bones or mediastinal organs. The PTV was created by adding a 2-mm margin to the CTV. For lesions visible only to one X‑ray camera (1-view cases), an asymmetrical CTV-to-PTV margin was applied of 2 mm in trackable and 4 mm in non-trackable projections. It should be noted that 0‑view (= ITV of the complete movement range of the tumor without motion tracking) was not used to avoid potential geometrical miss, as the CK system has no cone-beam CT. Thus, the radiotherapy treatment of patients ineligible for fiducial-free tumor tracking was transformed to LINAC-based SBRT. The CK treatment plans were calculated with the Accuray Precision TPS, using VOLO ™ optimization and Monte Carlo recalculation. All CK treatments were performed with the CyberKnife® M6 Robotic Radiosurgery System (Accuray Inc., Sunnyvale, CA) using 6‑MV FFF static IMRT fields with multileaf collimators (MLC).

### Applied dose schemes and dose prescription

Applied dose schemes were defined in our institutional protocol and were identical for both LINAC and CyberKnife treatments, using risk-adapted doses of 45–60 Gy in 3 to 8 fractions (biologically effective dose ranging from 86 to 151.2 Gy BED_10_). A dose of 8 × 7.5 Gy was given in cases with proximity to the hilar structures or heart or with broad contact with chest wall; 5 × 12 Gy was applied to lesions near or next to the chest wall or great vessels; 3 × 18 Gy was given if there was no limitation concerning the organs at risk (OARs). In our protocol, dose reduction was allowed if needed in order to protect OARs at the discretion of the treating radiation oncologist, keeping the originally designed number of fractions.

Though treatment planning requires distinct software, Eclipse for LINAC and Precision for Cyberknife, which both have specific characteristics of optimization and prescription (i.e., dose prescription as a function of PTV coverage for LINAC and PTV-encompassing isodose line for CK), the physical doses to the PTV were comparable.

The dose constraints for the PTV for all plans and both techniques (LINAC or CK) were as follows: minimum of 99% of the PTV volume should be covered by 95% of the prescribed dose (V95% > 99%), and 90% of the PTV volume should be covered by 100% of the prescribed dose (V100%> 90%). The maximum dose was typically between 120 and 130% of the prescribed dose.

Organ at risk dose constraints were defined in our institutional protocol and based on the AAPM Task Group 101 report [20].

### Statistics

Data were analyzed using the Statistica software (version 10. StatSoft Inc., Tulsa, USA).

Local control (LC) was defined as the interval between the first day of radiotherapy and the date of local recurrence of the lung tumor or the most recent follow-up. Patients who died of any cause without local recurrence were censored. Local progression-free survival (LPFS) was defined as the interval between the first day of radiotherapy and the date of local recurrence of the lung tumor, death, or the most recent follow-up. Progression-free survival (PFS) was defined as the interval between the first day of radiotherapy and the date of disease progression (local recurrence, new lung lesions distant from the PTV, lymph node metastases, distant metastasis), death, or the most recent follow-up. Overall survival (OS) was defined as the interval between the first day of radiotherapy and the date of death from any cause or the most recent follow-up. Actuarial LC, LPFS, PFS, and OS rates were calculated using Kaplan–Meier method.

Univariate analysis was performed using the Cox–Mantel test in order to investigate the prognostic value of factors influencing LC, LPFS, PFS, and OS. Multivariate analysis was performed using the Cox regression method. All *p*-values < 0.05 were considered to be significant.

Toxicities were evaluated using the CTCAE 5.0 (Common Terminology Criteria for Adverse Events).

The study was supported by the Hungarian National Ethical Committee (ETT-TUKEB).

## Results

### Patients

Between March 2015 and February 2023, 401 patients underwent curative-intent lung SBRT for early-stage primary lung cancer. The median age was 70 years (range 44–90). The median follow-up time (FUP) was 32 months (range 2–104). Though one of the inclusion criteria was to have a minimum time interval between the start treatment and the closure of the database, some patients died during the first year after SBRT, resulting in some cases with a FUP shorter than 12 months.

Pathological confirmation by bronchoscopy or percutaneous biopsy was available in 37.4% (*n* = 150) of patients (adenocarcinoma *n* = 92, squamous cell carcinoma *n* = 48, non-small cell lung cancer *n* = 9, small cell lung cancer *n* = 1), while histology was unknown in 62.6% (*n* = 251). Altogether, 18-FDG PET/CT was part of the pretreatment diagnostic workup in 96.3% (*n* = 386) of the entire cohort, and for nonverified cases, 18-FDG-PET positivity was mandatory.

Patient and tumor characteristics are summarized in Table 1. The T‑stage distribution was as follows: T1a in 32 patients (8%), T1b in 179 (44.6%), T1c in 112 (27.9%), T2a in 67 (16.7%), and T2b in 11 (2.7%). As the different size groups were disproportional, especially tumors larger than 4 cm (T2b) were underrepresented, patients were divided into two groups for the statistical analysis (T1 a,b vs T1c, T2a,b).

**Table 1:** Patient and tumor characteristics (*n* = 401)

Characteristic		Number (%/range)
Age, median (years, range)	–	70 (44–90)
Gender	Male	211 (53%)
	Female	190 (47%)
Tumor stage	T1a (≤ 1 cm)	32 (8%)
	T1b (> 1 to ≤ 2 cm)	179 (44.6%)
	T1c (> 2 to ≤ 3 cm)	112 (27.9%)
	T2a (> 3 to ≤ 4 cm)	67 (16.7%)
	T2b (> 4 to ≤ 5 cm)	11 (2.7%)
Histology	Adenocarcinoma	92 (23%)
	Squamous cell	48 (12%)
	Other	10 (2.5%)
	Unknown*	251 (62.5%)
Location	Left upper lobe	89 (22%)
	Left lower lobe	64 (16%)
	Right upper lobe	153 (38%)
	Right middle lobe	20 (5%)
	Right lower lobe	75 (19%)
ECOG	0	39 (10%)
	1	338 (84%)
	2	24 (6%)

*ECOG* Eastern Cooperative Oncology Group performance status

*Treatment was based on 18-FDG-PET/CT diagnosis

### Treatment

Risk-adapted treatment prescription doses were applied as a function of tumor size and location, with a higher number of fractions and lower BED for more centrally located lesions or in the case of proximity to the chest wall. The number of patients treated with LINAC- and CyberKnife-based SBRT was 362 and 39, respectively. Three major dose fractionation schemes were used: 3 × 18 Gy, 5 × 12 Gy, and 8 × 7.5 Gy, which could be reduced in order to respect organ at risk constraints. Previous analysis has already shown that the fractional dose can be reduced safely after optimization without compromising plan quality [21]. The most frequently administered dose was 5 × 12 Gy (*n* = 172). Median and mean BED_10_ were 132 and 121 Gy, respectively. Median volumes of the GTV and PTV were 3.9 cc (range 0.3–85) and 26.2 cc (range 5–196). Additional dosimetric data are shown in Table 2*.*

**Table 2:** Treatment characteristics (*n* = 401)

*Dose scheme*	*Number (%)*	*BED (Gy)*
3 × 15 Gy	1 (< 1%)	112.5
3 × 17 Gy	2 (< 1%)	137.7
3 × 18 Gy	34 (8.5%)	151.2
5 × 10 Gy	30 (7.5%)	100
5 × 11 Gy	38 (9.5%)	115.5
5 × 12 Gy	172 (43%)	132
8 × 6.5 Gy	3 (< 1%)	85.8
8 × 7 Gy	4 (1%)	95.2
8 × 7.5 Gy	117 (29%)	105
*BED*_*10*_		
≥ 132 Gy	208 (52%)	
< 132 Gy	193 (48%)	
*Technique*	*Number (%)*	
LINAC	362 (90%)	
CK	39 (10%)	
*Target Volume characteristics*	*Median*	*Mean (range)*
GTV volume (cc)	3.9	7.4 (0.3–85.1)
CTV volume (cc)	12.4	18 (1.5–144.7)
PTV volume (cc)	26.2	34.7 (5–195.9)
PTV D98% (Gy)	58.4	56.9 (43.6–63.8)
PTV coverage (V95) %	99.7	99.4 (91–100)
PTV coverage (V100) %	95	94.9 (72.7–100)

*BED*_*10*_ biologically effective dose with α/β = 10, *GTV* gross tumor volume, *CTV* clinical target volume, *PTV* planning target volume, *PTV D98%* dose covering 98% of the PTV

### Outcomes

**Fig. 1:**
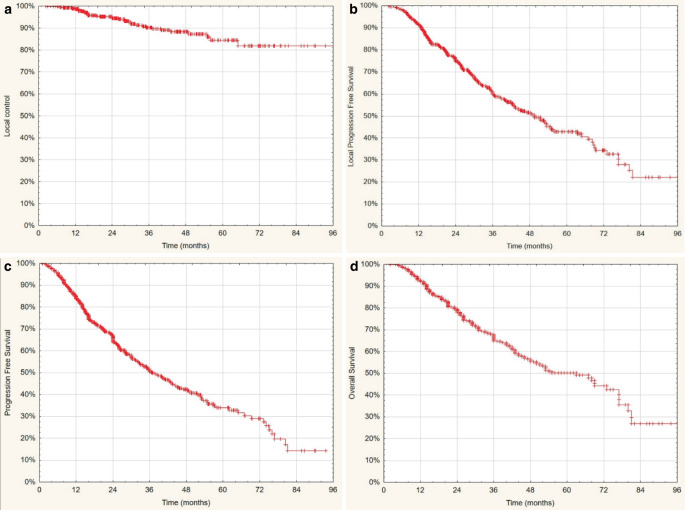
**a**–**d** Kaplan–Meier curves of the entire cohort of *n* = 401 patients treated with stereotactic body radiotherapy. **a** Local control, **b** local progression-free survival, **c** progression-free survival, **d** overall survival

After 32 months (range 2–104) of median follow-up, 233 of 401 patients (58%) were alive. In total, 36/401 patients (9%) developed local recurrence. Regional lymph node recurrence and out-of-field lung progression occurred in 33 (8.2%) and 60 cases (14.9%), respectively, while distant metastases developed in 72 patients (17.9%). Median OS was 63 months (95% CI: 51.1–74.8); 2‑, 3‑, and 4‑year OS was 79, 68 and 56%, respectively. The actuarial local control (LC) rate at 2 years was 94%, at 3 years 90%, and at 4 years 87%. The median was not reached. Median LPFS and PFS rates were 49.5 months (95% CI: 42.8–56.3) and 37 months (95% CI: 31.2.–42.8), respectively. Actuarial 2‑, 3‑, and 4‑year LPFS and PFS rates were 75, 60, and 51% and 66, 51, and 42%, respectively. Figure 1 shows the Kaplan–Meier curves of LC, LPFS, PFS, and OS for the entire cohort.

Univariate analysis of factors potentially correlated with survival endpoints included age (cut off at 70 years), gender, tumor stage (T1a, b vs. T1c, T2a,b), histological verification (proven vs. unknown), biologically effective dose with α/β = 10 (BED_10_), SBRT technique (LINAC vs. CK) and Eastern Cooperative Oncology Group (ECOG) performance status. In univariate analysis, factors positively affecting LPFS and OS were female gender (*p* = 0.001 and 0.005), smaller tumor size (*p* = 0.018 and 0.087), BED_10_ ≥ 132 Gy (*p* = 0.0046 and 0.03), and lower ECOG score (i.e., better ECOG performance status; *p* = 0.0044 and 0.0005). Significant advantages in terms of OS were also shown for BED > 105 Gy. The only factor affecting LC and PFS in univariate analysis was female gender, correlating with better survival rates (*p* = 0.005 and 0.001).

**Fig. 2:**
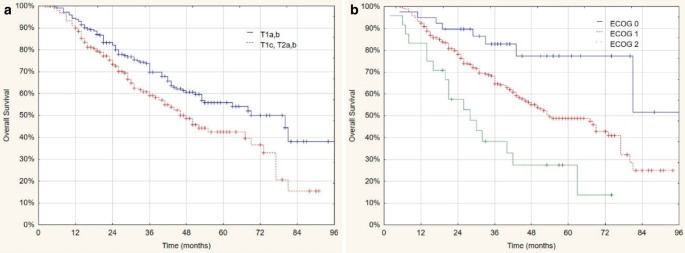
Kaplan–Meier curves of overall survival with variables from univariate analysis. **a** T stage 1a,b vs. T1c, T2a,b (*p* = 0.0044) and **b** ECOG performance status (*p* = 0.0005)

**Fig. 3:**
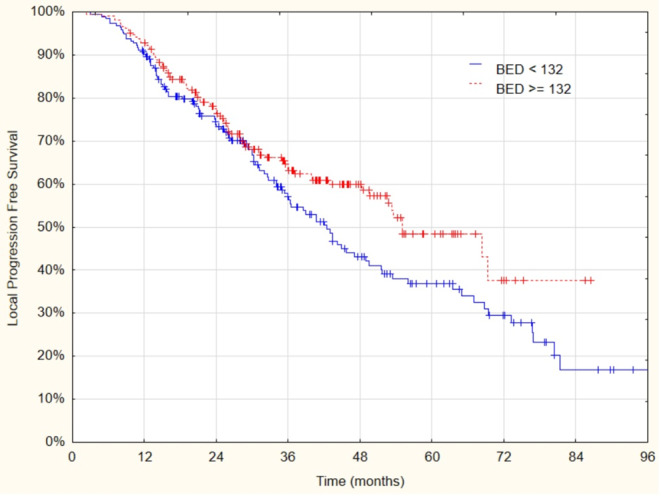
Kaplan–Meier curves of local progression-free survival as a function of biologically effective dose (*BED*) < 132 Gy vs. ≥ 132 Gy (*p* = 0.046)

In multivariate analysis, the model was highly significant for OS (*p* < 0.0001), and T stage (*p* = 0.002) and ECOG performance status (*p* < 0.001) arose as independent predictors, while BED_10_ ≥ 132 Gy was associated with better LPFS (*p* < 0.0001). Kaplan–Meier estimations for OS as a function of T stage and ECOG performance status are depicted in Fig. 2*,* and Kaplan–Meier estimations for LPFS as a function of BED dose are depicted in Fig. 3*.*

The median and 3‑year OS survival rates for T1a,b vs. T1c, T2a,b groups were 69 vs. 46 months (95% CI: 47.3–90.7 and 37–55) and 73 vs. 61% (*p* = 0.0044), respectively. The 3‑year OS rates for ECOG 0, 1, and 2 groups were 83, 68, and 37%, respectively (*p* = 0.0005).

The median and 3‑year LPFS rates for patients treated with BED_10_ doses of 132 Gy or higher vs. those with lower doses were 55 vs. 42.4 months (95% CI: 42.8–67.2 and 35.8–49) and 63 vs. 57% (*p* = 0.0046) respectively.

### Toxicities

Patients typically reported mild acute toxicities like fatigue, cough, or chest wall pain. No grade 3 or higher acute side effects were observed. The most common late side effect was grade 1–2 pulmonary fibrosis in 130 patients (32%). Grade 3 late side effects occurred in 4 patients (1%), including grade 3 late pulmonary fibrosis in 3 cases (0.7%) and potentially treatment-related grade 3 pneumothorax in 1 patient (0.2%; occurred 19 months after SBRT and was resolved by chest tube placement). Rib fracture was observed in 14 cases (3%). Mean time to the radiological diagnosis of rib fracture was 21 months (range 6–50). No late toxicities higher than grade 3 were observed. In our analysis, no correlation was found between the incidence of grade 3 toxicities or rib fracture and the prescribed dose (BED).

## Discussion

Stereotactic body radiotherapy became a standard treatment in the past decade, principally for the medically inoperable elderly population presenting early-stage NSCLC. We report a retrospective analysis of the long-term clinical outcome and safety of LINAC- and CK-based primary lung cancer SBRT in a leading tertiary comprehensive cancer center. The current work represents the largest cohort analysis published so far in Hungary on this subject and involves the first consecutively treated 401 patients since initiation of lung SBRT in our center.

Utilization of risk-adapted fractionation schemes in lung SBRT based on tumor size and relation to different OARs has already been validated for efficacy and safety by several workgroups [10, 22–25]. These reported experiences, alongside international guidelines on lung SBRT, helped us to establish our institutional lung SBRT protocol, with basically three dose-fractionation schemes (i.e., 3 × 18 Gy, 5 × 12 Gy, and 8 × 7.5 Gy). Using the strategy of individualized SBRT regimens, our analysis showed a high rate of estimated local control (90% at 3 years), which is consistent with previously reported studies. The 3‑year LC rates in published series reporting SBRT clinical results range between 77 and 96% [10–13]. Table 3 shows a comparative selection of studies comprising more than 100 patients.

**Table 3:** Series with *n* > 100 reporting results for early-stage primary lung cancer stereotactic body radiotherapy

Authors	Year	LINAC/CK	No. patients	Dose	LC (%)	OS (%)	Prospective/retrospective (P/R)	Single/multicentric (S/M)
Onishi et al. [14]	2007	LINAC	257	30–84 Gy (at isocenter) in 1–14 fx, median BED 111 Gy	LF*: 14%	3‑year: 56.8 5‑year: 46.2	R	M
Bahig et al. [15]	2014	CK	150	40–60 Gy in 3–5 fx, median BED 180 Gy	2‑year: 96	87	R	M
Davis et al. [39]	2015	LINAC/CK	723	10–80 Gy in 1–5 fx, median BED 151.2 Gy	1‑year: 88 2‑year: 76	1‑year: T1: 85, T2: 76 2‑year: T1: 63, T2: 52	R	M
Resova et al. [11]	2024	CK	172	30–60 Gy in 1–5 fx, median BED 151.2 Gy	1‑year: 97 2‑year: 95 3‑year: 90	84.9 NA 49	R	S
Stanic et al. [12]	2023	LINAC	206	34–60 Gy in 1–8 fx, median BED 115.5 Gy	1‑year: 98 2‑year: 96 3‑year: 96 5‑year: 95	87 74 62 31	R	S
Stephans et al. [29]	2017	LINAC	603	30–60 Gy in 1–10 fx	2‑year LF*: 13.1%	NA	R	S
Taremi et al. [25]	2012	LINAC	108	48–60 Gy in 3–10 fx	1‑year: 92 4‑year: 89	84 30	P	S
Temming et al. [10]	2018	CK	106	25–60 in 1–5 fx	2‑year: 88 3‑year: 77	77 56	R	S
Wegner et al. [13]	2018	LINAC	196	48–50 Gy in 4–5 fx, BED 100–105.6 Gy	3‑year: 94	58	R	S
Giuliani et al. [40]	2016	LINAC	734	18–64 Gy in 1–10 fx	1‑year: 98 2‑year: 94.4 5‑year: 91.7	82.1 63.7 31.8	R	M
*Current study*	2025	LINAC/CK	401	45–60 Gy in 3–8 fx, median BED 132 Gy	2‑year: 94 3‑year: 90 4‑year: 87	2‑year: 79 3‑year: 68 4‑year: 56	R	S

*LC* local control, *OS* overall survival, *LF* local failure, *BED* biologically effective dose with α/β = 10, *NA* not available, *LINAC* gantry-based linear accelerator, *CK* CyberKnife (Accuray, Madison, WI, USA), *fx* fractions

*LF local failure

In our series, the median OS was 63 months (95% CI: 51.1–74.8) and 3‑year OS was 68%, comparable to results in the relevant literature, with 3‑year overall survival rates reported between 55 and 87% [10, 12–16].

The question of the optimal dose in lung SBRT has been widely investigated, resulting in controversial conclusions. In earlier publications by Onishi et al. and Olsen et al., BED > 100 Gy was found to be associated with better LC and OS rates [14, 26]. Similarly, in a large multicentric pooled-cohort analysis of more than 500 lesions, Grills et al. reported that BED > 105 Gy predicts better local control [27]. Together with several other similar findings, these led to a widely accepted opinion that a threshold of BED 100–105 Gy is near to the optimal dose. However, in a meta-analysis of 34 observational studies on SBRT of stage I NSCLC comprising more than 2500 patients, Zhang et al. found conflicting results [28]. They investigated the prescribed dose divided into four groups—low (< 83.2 Gy), medium (83.2–106 Gy), medium-to-high (106–146 Gy), and high (> 146 Gy)—and the correlation with overall and cancer-specific survival (CSS) and local control rate. This study showed that patients treated with medium or medium-to-high BED (BED between 83.2–146 Gy) had higher OS than those treated in the low- or high-dose group.

More recently, Stephans et al., in a large, single-center analysis on a cohort of more than 600 patients, found that higher BED (150–180 Gy) was associated with lower rates of local failure, without a further benefit on OS [29].

Ruggieri et al. reported a critical review of the literature concerning the optimal dose and fraction number from a radiobiological point of view [30]. They concluded the existence of a dose–response relationship, with a saturation effect above a PTV-encompassing BED_10_ of 100–140 Gy, depending on the tumor volume. These results define quite a wide range, and it seems that the optimal SBRT dose is still not clear and probably is not uniform for every case, supporting risk-adapted fractionation.

One of the most important findings of our study is that a prescribed BED_10_ of 132 Gy or higher was associated with higher LPFS compared to the lower-dose group (median 55 vs. 42 months and 3‑year LPFS rate 63 vs. 57%); however, this advantage did not transform into better OS. This lack of correlation between fractionation regimens and OS but the presence of a positive relationship between higher BED_10_ and a local effect (LPFS) was most similar to findings of Stephans et al. [29], though the dose threshold was lower in the current study. Though our analysis was retrospective and heterogenous in many respects, our results may contribute to the body of clinical evidence in terms of the optimal lung SBRT dose. Our results emphasize the potential benefit of application of higher SBRT doses when it does not compromise organ at risk constraints.

With regards to OS, multivariate analysis showed a significant advantage for smaller tumors (T1a,b vs. T1c, T2a,b), and we found that treatment in an earlier tumor stage was associated with an almost 2‑year (23 months) median OS survival benefit, which underscores the importance of early diagnosis, even in an elderly, inoperable population. A similar correlation was shown by Fischer-Valuck et al., observing improved overall survival for tumors ≤ 30 mm compared to T2 (> 30 mm) tumors (*p* = 0.046) [31].

Taking into account that SBRT is generally performed in an elderly patient population, presenting severe comorbidities and poor lung function, international guidelines support the application of this therapy even for those who have contraindications to invasive biopsy. Several workgroups with a rate of unknown histology ranging between 26 and 65% and focusing on direct comparison of lung SBRT between biopsy proven and empirically treated (= clinical diagnosis only) groups also did not find significant differences in terms of survival, local effectiveness, or toxicity [13, 24, 25, 32–36]. In one of the largest cohorts, Verstegen et al. analyzed 591 patients (209 biopsy proven vs. 382 with clinical diagnosis). They did not observe significant differences between the two groups in terms of overall survival (*p* = 0.99) or local control (*p* = 0.98), and regional and distant recurrence rates were also similar [24]. Most recently, Fan et al. found an absence of significant differences between the two cohorts: 5‑year LC, PFS, CSS, and OS of 87 versus 83% (*p* = 0.58), 48 versus 45% (*p* = 0.82), 87 versus 84% (*p* = 0.65), and 60 versus 63% (*p* = 0.79), respectively. Recurrence patterns and toxicity were also similar [33]. Park et al. investigated SBRT in a group with purely unknown histology, concluding that empiric stereotactic radiotherapy in presumed early-stage NSCLC appears to be safe and may increase overall survival [37].

Altogether, these findings support the hypothesis that most patients with a correct clinical diagnosis of early-stage lung cancer indeed present malignant disease and will therefore benefit from a local ablative treatment approach like SBRT.

Similar to the abovementioned studies, in our analysis, no statistically significant difference was found in LC, LPFS, PFS, and OS between patients with or without pathological confirmation. Our results in a large patient population contribute to the findings reported in the literature concerning application of SBRT even if percutaneous biopsy is absolutely contraindicated.

Although our analysis was retrospective and was not designed to compare two techniques, we did not observe any difference between gantry-based LINAC and robotic (CK) SBRT. Although the latter two groups were quantitatively disproportional, the applied doses were similar; therefore, this finding might validate the parallel use of both techniques in the same institution, allowing clinicians to find the modality that best fits the lesion and patient characteristics.

We found the ECOG status to be the most important independent predictive factor of OS (*p* < 0.001) in multivariate analysis. When considering SBRT as a treatment for early-stage lung cancer, it is generally because of medical inoperability in patients with reduced life expectancy. Klement et al. focused on potential factors associated with early death after lung SBRT. Similarly to our findings, they observed that ECOG status and operability were the strongest predictors of early death (within 6 months after SBRT), and the Charlson comorbidity index was associated with the overall survival time [38]. These findings emphasize that despite the application of novel radiotherapy techniques and efforts searching for the optimal SBRT dose, the initial performance status remains by far the most important factor for survival in this elderly, inoperable population. These results once again underscore the importance of defining treatments with the highest rate of local control but still having the lowest incidence of severe side effects.

In general, SBRT treatments were well tolerated, and the rate of late grade 3 toxicity was very low, similar to published data (range 0–5.4%). Likewise, the frequency of rib fracture (3%) in our cohort was comparable to that in other studies (0–4%) [10–15].

The main limitations of the current study include its retrospective and single-institutional nature. This led to disproportional subgroups (tumor size, histology, dose, technique etc.) and may cause statistical biases. In our cohort, the rate of patients without histological confirmation was high (62.5%), which limits the interpretation of our results in biopsy proven NSCLC; however, the application of SBRT in such cases is supported by the literature, as discussed above. On the other hand, our findings support routine clinical decision-making in cases where surgery and invasive biopsy are contraindicated, yet a safe and potentially curative alternative is still required. It should be noted that due to retrospective data collection, the real incidence of toxicities might be underestimated. For the same reason, exact cause of death was not clear for a substantial number of patients, which was the reason why cancer-specific survival was not calculated. Another unique feature is that the follow-up period significantly overlapped with COVID-19 pandemic, potentially worsening OS rates in this pulmonary fragile population compared to series outside that interval. However, the large patient cohort and analysis of unselected, consecutive patients, thus leading to real-life results, are important strengths of our study. Furthermore, applied dose-fractionation regimens, dose prescription, and survival reporting were performed conforming to international guidelines and the related literature, thus enabling reliable comparison with previously published studies.

## Conclusion

The results of the current study on lung SBRT demonstrate outstanding long-term local control rates and promising LPFS, PFS, and OS rates, alongside a low incidence of severe toxicity, in elderly medically inoperable patients presenting with early-stage lung cancer. Our findings support the application of risk-adapted individualized fractionation, with a potential benefit for local progression-free survival using biologically effective doses above BED_10_ 132 Gy. Further studies are needed to investigate the question of the optimal SBRT dose.

## Supplementary Information

Tables summarizing results of Kaplan-Meier analysis (UNIVARIATE) and Multivariate analysis
