# Supplementary material for: Long-term clinical results of early-stage lung cancer patients treated with risk-adapted stereotactic body radiotherapy using LINAC or CyberKnife: A single-institution analysis of more than 400 cases
Source: Strahlenther Onkol. 2025 Aug 25;201(11):1208–18. doi: 10.1007/s00066-025-02455-3 (PMC12546400; doi:10.1007/s00066-025-02455-3)
Supplement: Supplementary file 1 — Tables summarizing results of Kaplan-Meier analysis (UNIVARIATE) and Multivariate analysis [file 66_2025_2455_MOESM1_ESM.docx]

|  | **LC** | **LPFS** | **PFS** | **OS** |
| --- | --- | --- | --- | --- |
| **Age** (<70 vs >70 years) | NS | NS | NS | NS |
| **Sex** (male vs female) | **p=0.005** | **p=0.001** | **p=0.0008** | **p=0.0087** |
| **T-stage** (T1a,b vs T1c,T2a,b) | NS | **p=0.018** | NS | **p=0.0044** |
| **Histology** (proven vs unkown) | NS | NS | NS | NS |
| **BED_10_** (<132 Gy vs ≥ 132 Gy | NS | **p=0.0457** | NS | **p=0.03** |
| **Technique** (LINAC vs CK) | NS | NS | NS | NS |
| **ECOG** (0 vs 1 vs 2) | NS | **p=0.0044** | NS | **p=0.0005** |

Summary of Kaplan-Meier analysis results (UNIVARIATE)

NS: non-significant, p-values below 0.05 were considered to be significant

Summary of results of Multivariate analysis

|  | **LPFS** |  | **OS** |
| --- | --- | --- | --- |
| **Modell** | **<0.0001** | **Modell** | **<0.0001** |
| **Sex** (male vs female) | **p=0.033** | **Sex** (male vs female) | NS |
| **T-stage** (T1a,b vs T1c,T2a,b) | **p=0.001** | **T-stage** (T1a,b vs T1c,T2a,b) | **0.002** |
| **BED10** (<132 Gy vs ≥ 132 Gy | **<0.0001** | **BED10** (<132 Gy vs ≥ 132 Gy | NS |
| **ECOG** (0 vs 1 vs 2) | NS | **ECOG** (0 vs 1 vs 2) | **<0.001** |
